# Off-label Sodium Oxybate in Childhood Narcolepsy: A Comprehensive Report

**DOI:** 10.7759/cureus.2526

**Published:** 2018-04-24

**Authors:** Jeffrey A Miskoff, Moiuz Chaudhri

**Affiliations:** 1 Medicine, Jersey Shore University Medical Center, Neptune City, USA

**Keywords:** case report, narcolepsy, cataplexy, orexin/hypocretin, sleep disorder, multiple sleep latency test, nocturnal polysomnogram, sodium oxybate, excessive daytime sleepiness

## Abstract

Narcolepsy is a sleep disorder that can manifest in childhood or adolescence by causing excessive sleepiness, hallucinations, sleep attacks, or cataplexy. There is often a significant delay in diagnosis with the mean time being 15 years from the onset of symptoms, which may lead to further exacerbations and a high comorbidity burden. Although narcolepsy is predominantly associated with loss of hypocretin (orexin), the role of genetics is poorly understood and, therefore, is complementary to the diagnosis but not confirmatory. We present the case of a child who was misdiagnosed as suffering from schizophrenia only to later uncover narcolepsy with cataplexy. Even though she did not meet strict criteria for narcolepsy type 1, her history and objective data were consistent enough to make an official diagnosis. In addition, her clinical response to treatment was very positive, further supporting narcolepsy as the most likely underlying condition. This presentation highlights the importance of continued education and research to reduce the risk of delay in diagnosis or misdiagnosis.

## Introduction

Narcolepsy is a complex sleep disorder that impairs the regulation of the sleep-wake cycle. Most patients present with excessive daytime sleepiness (EDS) occurring in isolation or in combination with features of abnormal rapid eye movement (REM), such as cataplexy, hypnagogic hallucinations, and sleep paralysis [1]. Narcolepsy is one of the most common causes of EDS despite being underdiagnosed due to lack of clinical experience [2]. Some studies have estimated the prevalence and incidence of narcolepsy to be 25 to 50 per 100,000 and 0.74 per 100,000, respectively [1-2]. Although the mechanism and etiology of this condition are not clear, evidence points toward a low level of hypocretin as the main culprit of this chronic condition [2].

## Case presentation

A six-year-old-African American female presented to our care in April 2010 for the evaluation of sleep attacks and apnea during sleep. According to her mother, the patient experienced cataplexy episodes with laughter. At that time, the patient underwent a nocturnal polysomnogram (NPSG) for further investigation. According to the results of the sleep study, patient slept 474.00 minutes out of 539.3 minutes in bed for a sleep efficiency of 87.9% (n=89%). Her sleep latency was decreased at 7.3 minutes and 68.1% of the total sleep time was spent in the supine position. In addition, rapid eye movement (REM) sleep and latency were logged at 17% and 68.5 minutes (n=136-156), respectively. Furthermore, the NPSG illustrated that the patient experienced five central apneas, one mixed apnea, five hypopneas, and an apnea-hypopnea index of 1.4 events/hour consistent with mild obstructive sleep apnea (OSA) by pediatric criteria [1].

The patient underwent another NPSG with a multiple sleep latency test (MSLT) in November 2010 for persistent symptoms. Results indicated that the patient slept for 395.50 minutes out of the 411.8 minutes in bed, which translated to a sleep efficiency of 96.0%. Similar to the previous study, the patient experienced decreased sleep latency at 3.3 minutes. In addition, REM sleep and latency were logged at 20.4% and 106.5 minutes, respectively. In contrast to the earlier NPSG study, this evaluation illustrated three central apneas, six mixed apneas, nine obstructive hypopneas, and an apnea-hypopnea index of 2.9 events/hour with <1 being normal.

An MSLT is the primary diagnostic tool for narcolepsy and is typically performed following an NPSG to measure sleep latency, which is the time it takes an individual to go from wakefulness to sleep. Additionally, it measures how quickly the patient enters REM sleep, known as sleep onset REM periods (SOREMPs). This test includes four or five nap periods of 20 minutes each [3]. The patient is instructed to lie in the darkroom and attempt to fall asleep. Sensors transmit data such as the amount of time needed to fall asleep and enter REM sleep. Once asleep, the patient will be woken up fifteen minutes later; if 20 minutes pass and the patient has not fallen asleep, the nap trial is terminated. Next, the patient is given a two-hour break before the second nap trial can begin. During this break, the patient must stay awake and this process is repeated four more times [3]. The results of the study include the mean sleep latency (MSL) and the number of SOREMPs recorded. The diagnostic criterion requires an MSL of ≤8 minutes and ≥2 SOREMPs on an MSLT [1]. Our patient underwent a series of five nap trials with a sleep latency of 3, 2, 3, 6, and 2 minutes, respectively, and a mean sleep latency of 3.2 minutes. However, the patient did not enter REM sleep during any nap trials, thus having 0 SOREMPS. Additionally, laboratory workup for HLA-DR15 and DQ0602 was positive, and the patient had documented cataplexy.

## Discussion

Narcolepsy is one of the most common causes of excessive daytime sleepiness whose etiology is multifactorial. Although the role of genetics and rare brain lesions cannot be minimized, the loss of hypocretin is highly associated with the development of this condition [4]. Evidence suggests that hypocretin (an endogenous ligand for a G protein-coupled receptor) is a neuropeptide found in perifornical, lateral, and posterior hypothalamus targets monoaminergic areas (i.e. locus coeruleus, raphe nuclei, and tuberomammillary nucleus) and cholinergic areas (i.e. basal forebrain and laterodorsal tegmental nuclei) to increase activity, promoting wakefulness, inhibiting REM sleep, and suppressing REM phenomena, such as cataplexy [4]. Research indicates that low levels of hypocretin in type I narcolepsy is primarily due to the destruction of hypocretin-producing neurons in the hypothalamus [4]. Additionally, an autoimmune phenomenon is thought to be the driving force behind the destruction of hypocretin neurons in the hypothalamus.

Narcolepsy can be grouped into type 1 (narcolepsy with cataplexy) and type 2 (narcolepsy without cataplexy). Although excessive daytime sleepiness is always present in narcolepsy, this symptom does not differentiate between type 1 or type 2 narcolepsy [1]. However, a positive finding of cataplexy is considered pathognomonic for the diagnosis of narcolepsy because it is rarely found in any other disorder [1,3]. Although clinical manifestations may vary, the patient experiences the REM disorder. Patients often struggle with the ability to fall asleep at night while others fall asleep suddenly regardless of the situation. In contrast, some patients fall asleep suddenly even if they are engaged in activities such as talking and eating [3-4]. Narcolepsy usually presents between the ages of 10 to 20 years with the sudden onset of excessive daytime sleepiness. However, it can also present as early as the age of five years [1,4]. Early onset narcolepsy can present with prominent oculobuccofacial involvement, irritability, and hyperactivity [2].

We presented a six-year-old female who presented to our care in April 2010 for an evaluation of sleep attacks, snoring, and hypnopompic/hypnagogic hallucinations dating back approximately two years. Over the years, the patient experienced frequent cataplexy episodes triggered by laughter. Additionally, the child experienced the sensation of spiders crawling on her arms upon awakening and described additional visual hallucinations of people sitting on her bed upon falling asleep. These complaints led her to be diagnosed with schizophrenia. Additionally, per the patient's mother, other conditions, such as bipolar disorder and psychosis, were also considered.

Current diagnostic criteria for type 1 narcolepsy require the patient to meet both of the following: (1) Excessive daytime sleepiness (EDS), occurring for at least three months; (2) One or both of the following: (a) Cataplexy, mean sleep latency of ≤8 minutes and ≥2 SOREMPs on an MSLT; a SOREMP on the preceding NPSG (REM onset within 15 minutes of sleep onset) may replace one of the SOREMPs on the MSLT; and (b) Cerebrospinal fluid (CSF) hypocretin-1 levels less than ≤110 pg./ml [1,3]. Guidelines state that the mean sleep latency of ≤8 minutes on an MSLT is abnormal and latencies <5 minutes indicate severe EDS [1]. Our patient exhibited excessive daytime sleepiness, cataplexy, and mean sleep latency of 3.2 minutes; however; no SOREMPs were noted on the MSLT. Despite falling short of satisfying the SOREMP component of the diagnostic criteria, her clinical picture, when viewed as a whole, was still found to be consistent with the diagnosis of narcolepsy.

Although narcolepsy is predominantly associated with loss of hypocretin, the role of genetics is less well understood and, therefore, is complementary to the diagnosis but not confirmatory. Evidence suggests a strong association between narcolepsy and human leukocyte antigen (HLA) especially HLA-DQB1*0602 [5]. The positivity of DQB1*0602, a subtype of DR2, in African American patients has been linked with the earlier onset of narcolepsy [5]. Since the patient tested positive for this gene, this further supports the diagnosis.

Narcolepsy is a lifelong condition managed with a combination of pharmacologic and non-pharmacological agents. Pharmacologic agents are tailored to the individual based on the symptoms and severity. Current agents, such as amphetamines, modafinil, and antidepressants focusing on inhibiting norepinephrine or serotonin reuptake, are effective in reducing sleepiness and cataplexy [1,5].

Sodium oxybate (Xyrem) is the sodium salt of a compound, γ-hydroxybutyric acid (GHB), which serves as a precursor of the neurotransmitter γ-aminobutyric acid (GABA) along with possessing neuromodulator properties, and is involved in sleep regulation [6]. Furthermore, clinical evidence suggests that this compound is highly effective and safe in the treatment of narcolepsy due to its natural properties [6]. A study conducted in 1997 focused on 48 narcoleptic patients who were administered 2.25 to 3 grams of GHB daily for nine years [7]. Results indicated that this treatment alleviated their symptoms significantly without developing tolerance or addiction. It has been proposed that GHB induces the hyperdopaminergic process by modifying acetylcholine release [7]. Moreover, the role of GHB in increasing stages 3 and 4 sleep along with decreasing stage 1 non-REM (NREM) sleep is supported by decades of clinical evidence [1,7].

Although Xyrem was approved by the FDA for narcolepsy in patients 18 years of age and older, a decision by the treating clinician to start the therapy off-label at a reduced dose was made in attempts to control both the cataplexy and severe hypersomnia complaints despite the patient only being seven years of age. To date, the patient remains on a reduced dose of Xyrem at 2.5 g at bedtime and repeated three to four hours later. Typically, in adults, Xyrem is started at 2.25 g at bedtime and repeated three to four hours later due to a short half-life (two to three hours) [8]. Recommendations suggest the need to titrate to a max dose of 4.5 g over approximately four weeks if the symptoms persist.

The patient was ultimately started on 2.5 mg Adderall in the morning as her first dose. As the patient progressed, the morning dose was increased along with adding an additional dose in the afternoon to control the symptoms. Current recommendations favor 5 mg in the morning as being the starting dose of amphetamines in children [6,8]. Furthermore, recommendations suggest 10 to 40 mg per day divided into two doses as being effective for children [8]. As of October 2017, the patient is on 20 mg Adderall taken in the morning along with an additional dose of 20 mg to be taken in the afternoon as needed. According to her mother, the patients’ symptoms are well controlled, and she is excelling in school, indicated by her making the honor roll and earning all As.

Although pharmacologic management is at the forefront, behavioral and lifestyle modifications play a crucial role in reducing the impact of the disease. Modifications such as scheduled naps, practicing good sleep hygiene, and regular exercise during the day to improve alertness, being cautious around moving equipment/machinery and heights are beneficial. Interestingly, studies indicate that regulation of body temperature can be an important tool in the management of this condition. Specifically, higher body temperature is associated with improved vigilance, which can be used by the patients to improve their condition [9].

Undoubtedly, existing medications are effective for the symptomatic management of this condition; research has illustrated histamine agonist and hypocretin analogs, which show promise. Pitolisant, an inverse agonist of the histamine H3 receptor, has shown to decrease cataplexy and improve wakefulness in patients with type 1 narcolepsy [10]. It has been granted orphan drug status by FDA, but it is only available in Europe and not being sold in the United States [10].

## Conclusions

In conclusion, there are cases of pediatric patients misdiagnosed as having psychiatric disorders that may actually represent narcolepsy with cataplexy as in our patient. Even though she did not meet strict criteria for narcolepsy type 1, her history and objective data were strong enough to make an official diagnosis. In addition, her clinical response to medications was excellent, further cementing her underlying condition. Finally, staying abreast of new Food and Drug Authority (FDA)-approved medications despite effective treatment options is critical to improving medical care.
